# Detecting Human Epidermal Growth Factor Receptor 2 (HER2) Amplification: Proof of Concept of an Alternative Approach

**DOI:** 10.7759/cureus.44785

**Published:** 2023-09-06

**Authors:** Shikha Mudgal, Pranoy Paul, Bina Ravi, Shruti Agrawal, Arnav Kalra, Shalinee Rao, Nilotpal Chowdhury

**Affiliations:** 1 Pathology and Laboratory Medicine, All India Institute of Medical Sciences, Rishikesh, Rishikesh, IND; 2 Integrated Breast Care Centre, All India Institute of Medical Sciences, Rishikesh, Rishikesh, IND; 3 General Medicine, All India Institute of Medical Sciences, Rishikesh, Rishikesh, IND

**Keywords:** pgap3, grb7, erbb2, immunohistochemistry, breast carcinoma, breast neoplasms

## Abstract

Background: There are multiple genes that are co-amplified along with human epidermal growth factor receptor 2 (HER2) in chromosome 17. GRB7 and PGAP3 are two such genes. We hypothesize that the protein products of these genes may serve as immunohistochemistry (IHC) markers for detecting HER2 amplification in breast cancer.

Methods: Tissue sections from one hundred and thirty-five primary breast carcinoma cases were subjected to immunohistochemical staining for antibodies against HER2, GRB7, and PGAP3 and graded on a scale of 1 to 3. Both membranous staining and cytoplasmic staining were assessed for GRB7 and PGAP3. For equivocal HER2 IHC positivity, fluorescent in situ hybridization was performed to get the final HER2 status.

Results: IHC staining for GRB7 and PGAP 3 was a moderate to strong predictor for HER2 status (area under the curve (AUC) of 0.768, 0.868,0.754, and 0.790 for GRB7 membranous staining, GRB7 cytoplasmic staining, PGAP3 membranous staining, and PGAP3 cytoplasmic staining respectively). A combination of GRB7 cytoplasmic and PGAP3 membranous staining resulted in an AUC of 0.905 (95% CI 0.855-0.954), while a combination of GRB7 and PGAP3 cytoplasmic staining resulted in an AUC of 0.902 (95% CI 0.851-0.953).

Conclusion: The point estimates for the AUC of GRB7 and combined GRB7 and PGAP3 in predicting the AUC suggest a strong predictive ability of these markers to predict HER2. With further refinement in technique, cytoplasmic staining and membranous IHC staining for GRB7 and PGAP3 have potential to serve as surrogate markers for HER2 status. The strategy of using protein products of co-amplified genes of HER2 is likely to be successful in technical validation.

## Introduction

Human epidermal growth factor receptor 2 (HER2) amplification testing is an essential part of breast cancer workup [1]. HER2-amplified cancer patients are eligible for anti-HER2 therapy by trastuzumab or lapatinib [1]. Besides this, HER2 amplification is also a prognostic factor for breast cancer and is a part of the recent AJCC staging. The amplification is primarily tested by immunohistochemistry (IHC) for overexpression complemented by in situ hybridization (ISH) for cases that are equivocal on IHC [2]. ISH requires technical expertise and is about ten times costly compared to IHC in our setup. In such cases, it would be convenient to have alternative markers for IHC which will be able to play a complementary role in breast cancer testing.

The HER2 amplification region on chromosome 17 contains other genes that are co-amplified [3-5]. These genes have been shown to be strongly co-expressed with HER2 in micro-array studies. Examples of such genes include growth factor receptor bound protein 7 (GRB7), post-GPI attachment to protein phospholipase 3 (PGAP3), StAR-related lipid transfer Domain containing 3 (STARD3), and cyclin-dependent kinase 12 (CDK12). Independent studies have shown that these markers are strongly associated with HER2, but their performance as diagnostic predictors of HER2 status has not been studied [6-8]. In this study, we will evaluate the role of two such genes (GRB7 and PGAP3) [9,10]. Our hypothesis is that these genes or their protein products, singly or in combination, are suitable as alternative markers for diagnosing HER2 status. This is a proof-of-concept study designed to demonstrate feasibility, which, if found successful, will lead to further refinement of technique and standardization and lead to the establishment of a diagnostic test. Furthermore, this may also be treated as demonstration of in-house quality control for IHC staining for HER2.

## Materials and methods

Our study was approved by the Institutional Ethics Committee of All India Institute of Medical Sciences, Rishikesh. One hundred and thirty-five breast cancer cases were selected, and their core-biopsies and blocks were retrieved. The IHC status of these cases was confirmed with respect to their IHC and/or ISH status. Of these cases, two were excluded (one case was an IHC 2+ (equivocal) tumor not having an ISH report, and another one did not have sufficient material for further testing). Sections were cut from these blocks and then immuno-stained with GRB7 antibodies (make Elabsciences, Catalogue no. E-AB-14108) and PGAP3 antibodies (make Abbexa, Catalogue no. abx027296 and ThermoFisher, Catalogue no. PA5- 53451). GRB7 staining failed in one case, which was removed for statistical analysis involving GRB7 staining as one of the variables. PGAP3 staining failed in a separate case, which was similarly removed for statistical analysis involving PGAP3 staining. 

The HER2 antibodies (make Quartett, clone QR-003) were evaluated according to commonly followed guidelines [2]. The IHC for HER2 was classified into four categories, 0 (no staining), 1+ (incomplete faint membrane staining), 2+ (complete faint membrane staining in more than 10% of tumor cells or strong complete membrane staining in less than 10% of tumor cells), or 3+ (string complete membrane staining in more than 10% of tumor cells). 0 and 1+ stains were classified as negative, 2+ as equivocal and 3+ as positive for HER2. The equivocal cases were subjected to dual-color fluorescent-in situ-hybridization (FISH) of make FlexISH ERBB2/CEN17 Dual color probe, from Zytovision, Zytomed, Berlin, Germany. FISH was classified as positive if the CEN17/HER2 ratio was greater than 2.0 or if the HER2 count was more than 6.0 on average. The ASCO/CAP guidelines for reporting ISH were followed [11].

**Figure 1:**
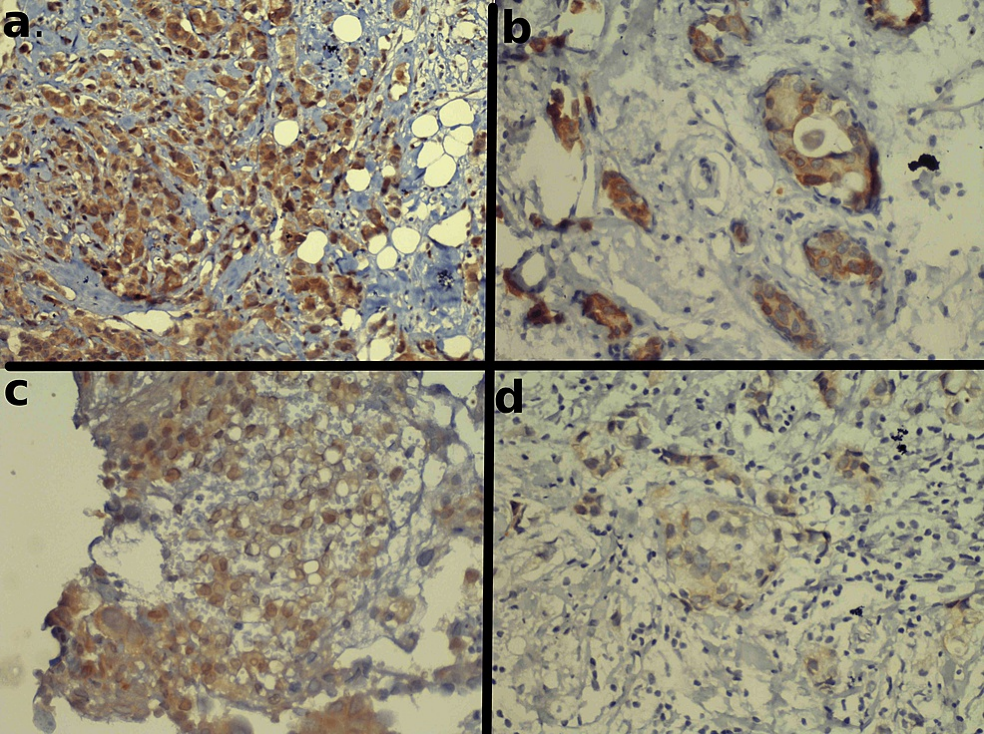
Representative pictures of grades applied to cytoplasmic IHC staining. a: Section showing diffuse, strong, granular, grade 3 cytoplasmic positivity (magnification, 100x) for antibody to GRB7, b: Grade 2 cytoplasmic positivity, with a mixture of strong and weak staining patterns in almost equal proportion for PGAP3, c: Weak non-granular stain involving most of the cells (grade 1 cytoplasmic positivity) for GRB7. D. Negative (grade 0) cytoplasmic staining, with most cells showing no staining, or only weak patchy stain in a minority of the cells.

For GRB7 and PGAP3, both cytoplasmic positivity and membranous positivity were noted separately. Membranous positivity was interpreted like IHC for HER2. Cytoplasmic evaluation was done subjectively by categorizing cases as 0 (negative), 1 (mild), 2 (moderate), or 3 (marked). A tumor was graded as cytoplasmic grade 0 if there was no staining or weak, non-granular, staining in a minority of the cells. Grade 1 staining was defined as a weak, non-granular staining affecting most of the cells. Grade 2 staining was defined as the presence of a mixture of weak, non-granular cytoplasmic staining as well as strong granular staining in most of the cells. Grade 3 cytoplasmic staining was defined as the presence of strong, granular staining in most of the cells. Representative pictures of cytoplasmic staining grading are given in Figure 1.

The association between HER2 status and GRB7/PGAP3 was assessed by the Fisher exact test, logistic regression, and receiver-operating-characteristic (ROC) curve analysis. The area under the ROC curve (AUC) was calculated with the 95% confidence interval (CI) by the DeLong method. By multiple logistic regression and stepwise selection based on the Akaike Information Criterion (AIC), the best independent predictors of HER2 status were selected. Finally, as a simple model, the scores of the independent predictors were summed, and the diagnostic performance of the summed variable was evaluated. The statistical calculations were done by R statistical environment version 4.1.1 along with the package “pROC” and the R commander plugin EZR [12,13].

## Results

The clinical characteristics of the cancer samples studied are given in Table 1.

**Table 1:** The clinical characteristics of the included patients. For age and size, the median and range are given. For the other variables, the count is given. ER: Estrogen receptor, PR: progesterone receptor, HER2: human epidermal growth factor receptor 2, FNAC: fine-needle aspiration cytology

Variable	Characteristic described	Value
Age (yrs)	Median (Range)	48 (26 - 80)
Size (cm)	Median (Range)	3.1 (1.0 - 10.0)
Grade	1	4
	2	34
	3	85
	Not graded	10
ER	Positive	80
	Negative	53
PR	Positive	64
	Negative	69
HER2	Positive	71
	Negative	62
Lymph node	Positive on FNAC	68
	Lympadenopathy positive, FNAC negative	21
	Negative for lymphadenopathy	44

All four predictors (cytoplasmic staining of GRB7, membranous staining of GRB7, cytoplasmic staining of PGAP3 and membranous staining of PGAP3) were significantly associated with HER2 (Table 2). The cytoplasmic stains showed higher predictive strength than the membranous stains, with GRB7 showing the highest predictive strength.

**Table 2:** The relationship of the GRB7 and PGAP3 immunohistochemistry (IHC) staining with HER2 status. HER2: Human epidermal growth factor receptor 2, GRB7: growth factor receptor bound protein 7, PGAP3: post-GPI Attachment to protein phospholipase 3

Biomarker	Grade	HER2 Negative	HER2 Positive	Fisher's exact test p-value	Area under curve [95% CI]
GRB7-cytoplasmic	0	45	10	<0.0001	0.868 [0.811, 0.924]
	1	15	21		
	2	1	18		
	3	0	22		
GRB7-membranous	0	51	28	<0.0001	0.758 [0.688, 0.827]
	1	9	9		
	2	1	13		
	3	0	21		
PGAP3-cytoplasmic	0	55	23	<0.0001	0.790 [0.722, 0.857]
	1	4	17		
	2	2	13		
	3	1	17		
PGAP3-membranous	0	56	30	<0.0001	0.754 [0.687, 0.820]
	1	5	12		
	2	1	14		
	3	0	14		

On multivariate analysis, cytoplasmic staining with GRB7 and membranous staining with PGAP3 were independent of one another, with the AUC of the model showing a high AUC (Table 3).

**Table 3:** The results of multivariate logistic regression with the most parsimonious fit among the different models used to predict the human epidermal growth factor receptor 2 (HER2) status. GRB7 membranous and PGAP3 cytoplasmic immunohistochemistry staining were the other competitors. GRB7: Growth factor receptor bound protein 7, PGAP3: post-GPI attachment to protein phospholipase 3

Variable	Coefficient	Standard Error	Odds Ratio [95% Confidence Interval]	p-value
[Intercept]	-2.1057	0.4006	0.122 [0.056, 0.267]	<0.0001
GRB7-cytoplasmic	2.02	0.4028	7.54 [3.42,16.60]	<0.0001
PGAP3-membranous	1.3192	0.4463	3.74 [1.56, 8.97]	0.0031
Null deviance: 182.506 on 131 degrees of freedom, Residual deviance: 92.024 on 129 degrees of freedom. Area under curve: 0.910 [95% CI: 0.861, 0.959]				

The scores of cytoplasmic staining with GRB7 and membranous staining with PGAP3 were added to form a single variable. This summed variable (GRB7 cytoplasmic + PGAP3 membranous) also showed a high AUC suggesting strong predictive ability (AUC= 0.905; 95% CI: 0.856-0.955). The summed score of GRB7 cytoplasmic and PGAP3 cytoplasmic stain also suggested strong predictive ability with an AUC of 0.902 and 95% CI of 0.851-0.953. The diagnostic accuracy of the summed scores is given in Table 4.

**Table 4:** Area under the curves (AUCs) of the various immunohistochemical staining with the scores of GRB7 and PGAP3 added together, along with the sensitivity, specificity, and accuracy at the best cut-off. 95% confidence intervals are given in parentheses. GRB7: Growth factor receptor bound protein 7, PGAP3: post-GPI attachment to protein phospholipase 3

Combination (Sum) of variables	AUC [95% CI]	Best Cut-off Score	Sensitivity [95% CI]	Specificity [95% CI]	Accuracy [95% CI]
GRB7 cytoplasmic + PGAP3 membranous	0.905 [0.855, 0.954]	2	0.74 [0.62, 0.84]	0.97 [0.89, 1]	0.85 [0.78, 0.90]
GRB7 cytoplasmic + PGAP3 cytoplasmic	0.902 [0.851 ,0.953]	2	0.77 [0.66, 0.86]	0.94 [0.84, 0.98]	0.85 [0.78, 0.90]
GRB7 membranous +PGAP3 membranous	0.849 [0.786 ,0.912]	2	0.70 [0.58, 0.80]	0.97 [0.89, 1]	0.83 [0.75, 0.89]
GRB7 membranous + PGAP3 cytoplasmic	0.866 [0.807, 0.925]	2	0.73 [0.61, 0.83]	0.90 [0.80, 0.96]	0.81 [0.73, 0.87]
GRB7 cytoplasmic +GRB7 membranous	0.871 [0.814, 0.927]	1	0.90 [0.81, 0.96]	0.66 [0.53, 0.78]	0.79 [0.71, 0.86]
PGAP3 cytoplasmic +PGAP3 membranous	0.784 [0.714, 0.854]	2	0.57 [0.45, 0.69]	0.95 [0.87, 0.99]	0.75 [0.67, 0.82]

There were 20 HER2+ tumors on IHC. All twenty of them turned out to be negative on FISH. Hence, sensitivity could not be calculated in these cases due to the absence of true positive cases. However, the specificity could be calculated for GRB7, PGAP3, and different combinations of these biomarkers which is given in Table 5. The specificity of the markers studied were generally high.

**Table 5:** The specificity of a negative test result in IHC HER2 2+ tumors (n=20). For a single marker, grade 0 was taken as negative. For a combination of markers, the best cut-offs as shown in Table 4 were used.

Marker	Negative	Positive	Specificity [95% CI]
GRB7-cytoplasmic	11	9	0.55 [0.32, 0.77]
GRB7-membranous	16	4	0.80 [0.56, 0.94]
PGAP3-cytoplasmic	18	2	0.90 [0.68, 0.99]
PGAP3-membranous	19	1	0.95 [0.75, 1]
GRB7 cytoplasmic + PGAP3 membranous	18	2	0.90 [0.68, 0.99]
GRB7 cytoplasmic + PGAP3 cytoplasmic	17	3	0.85 [0.62, 0.97]
GRB7 membranous +PGAP3 membranous	19	1	0.95 [0.75, 1]
GRB7 membranous + PGAP3 cytoplasmic	18	2	0.90 [0.68, 0.99]
GRB7 cytoplasmic +GRB7 membranous	11	9	0.55 [0.32, 0.77]
PGAP3 cytoplasmic +PGAP3 membranous	18	2	0.90 [0.68, 0.99]

## Discussion

Both GRB7 and PGAP3 have been found to be significant predictors of HER2 status. Taken individually, the predictive strength is modest, with only cytoplasmic staining of GRB7 attaining an AUC of greater than 0.85. However, when the combined score of GRB7 and PGAP3 is taken, the prognostic strength increases markedly, attaining an AUC value of greater than 0.9. This itself is proof of the predictive strength of GRB7 and PGAP3 and suggests that with improvement in retrieval techniques, better prediction will be attained. This itself is not surprising since amplification of the HER2 locus leads to amplification of these genes as well, being present in the same amplicon [5]. GRB7 and PGAP3 should be seen as alternative sites for the detection of the same target rather than alternative different markers.

The main advantage of using IHC for different protein products of genes co-amplified with HER2 is that IHC is of an order of magnitude cheaper than ISH. In addition, ISH requires a specialized, more sophisticated laboratory and more expensive technical staff. IHC is therefore potentially cost-saving for those laboratories that have IHC but do not have FISH. Also, staining for GRB7 and PGAP3 may serve as quality control indicators of IHC staining in cases where there may be scarcity or non-availability of proper control tissue.

One additional takeaway of this study is that the cytoplasmic staining of GRB7 and PGAP3 may be more informative than membranous staining alone. Examination of the protein atlas suggests that GRB7 and PGAP3 are localized both in the cytoplasm as well as on the plasma membrane [9,10]. The sites of action of both are on the cytoplasmic domain of membrane receptors. This study therefore serves as evidence for the predominant active subcellular site of these two proteins, both of which play a key role in cancer biology and have prognostic implications [8,14,15].

It needs to be emphasized that this is a large proof-of-concept study. Due to the considerable number of cases, this can also serve as an initial validation study of GRB7 and PGAP3 in predicting HER2 status. These initial results are promising with a concordance approaching 85% but still have failed to demonstrate a concordance of greater than 95% with the reference standard when taking the optimal cut-off (Table 4). This is primarily due to the low sensitivity (less than 80%) of the assays. Such a low sensitivity resulting in a false negative result may be due to suboptimal dilution, epitope retrieval, or transcript level variability in the GRB7 and PGAP3 proteins. This suggests that further improvements in antibody concentration and epitope retrieval need to be done. Now that the proof-of-concept and initial validation have been provided, the next step would be to standardize the technique as per the temperature, buffer concentration, retrieval technique, antibody concentration, and, if needed, antibody selection (since there are six known transcript variants of each of the genes), followed by lot-to-lot reproducibility studies [16-18]. Automated IHC staining may help. We hope to continue such studies in the future. Validating these antibodies is also important since GRB7 and PGAP3 may themselves serve as important genes for tumor biology in breast and other cancers [8,14,15,19-21]. GRB7 may have additional therapeutic implications for targeted therapy [19,20].

The primary limitation of this study was that the method of assessment of cytoplasmic staining was subjective. During future validation, clearly defined criteria for cytoplasmic staining will be laid down during validation. Fine granular staining of the cytoplasm, coarse granular staining, and the extent of masking of nuclei are being investigated. Furthermore, detailed assessment of antibody clones and techniques is also required. Another limitation is that we could not assess the diagnostic accuracy in the subgroup of HER2 2+ (on IHC) tumors due to all such tumors being negative for HER2 amplification on FISH. The specificity of the markers in HER2 2+ tumors was generally high, but further validation of GRB7 and PGAP3 antibodies in a subset containing an adequate number of such HER2 amplified HER2 2+ tumors is needed after further standardization of technique. 

## Conclusions

This study has demonstrated strong predictive ability of GRB7 and PGAP3 in combination for detecting HER2 amplified breast cancers. The predictive ability should get only stronger with further standardization and refinement in technique. Both GRB7 and PGAP3 are possible and promising alternative biomarkers for detecting amplification of the HER2 related site on chromosome 17.
